# Supplementary figures and images for: TEsmall Identifies Small RNAs Associated With Targeted Inhibitor Resistance in Melanoma
Source: Front Genet. 2018 Oct 5;9:461. doi: 10.3389/fgene.2018.00461 (PMC6186986; doi:10.3389/fgene.2018.00461)

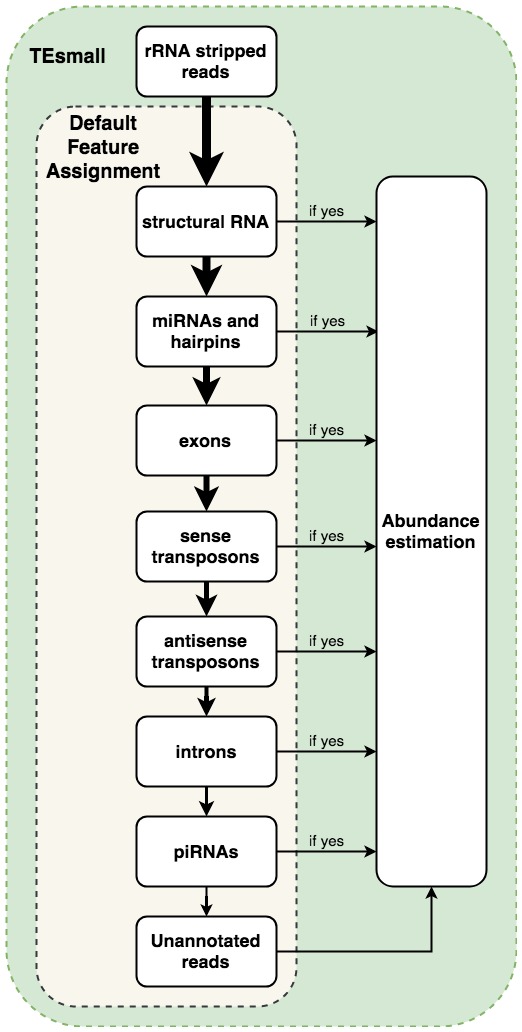

Supplement: FIGURE S1 — Flow chart of the default sequential decision tree used by TEsmall to assign annotations. Alignments are assigned to each category in the indicated order and, if annotated, are removed from the pool before preceding to the next annotation category. Users may opt to re-order the priority table. [file Image_1.JPEG]

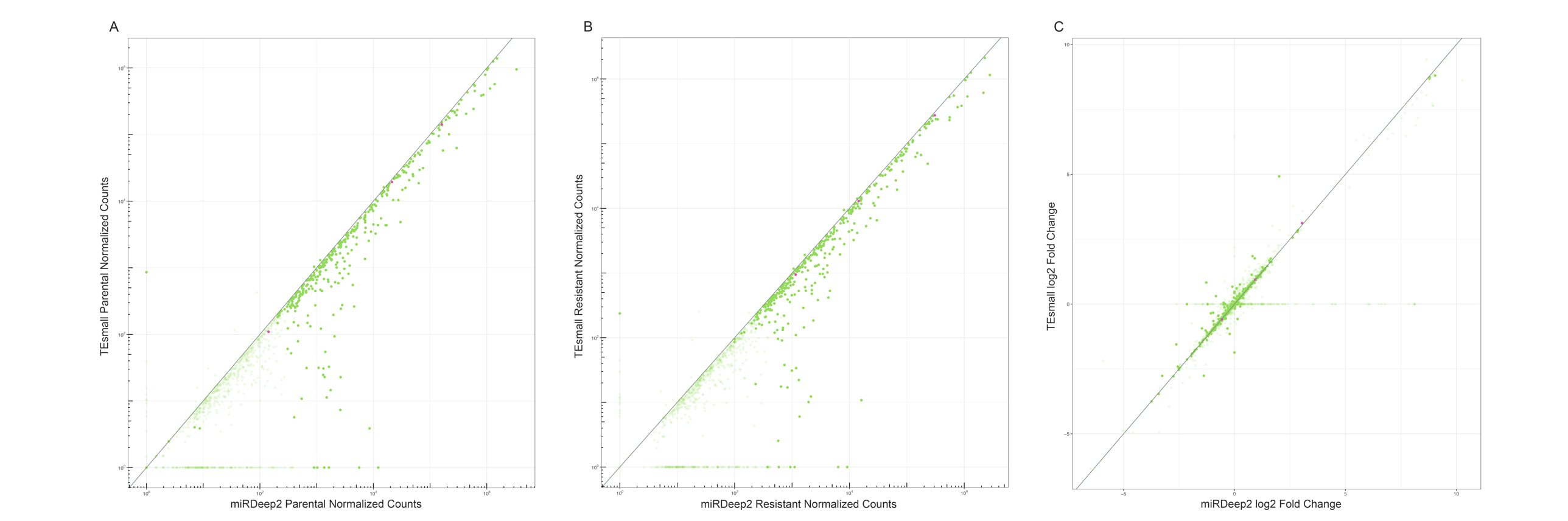

Supplement: FIGURE S2 — Scatterplots comparing TEsmall and miRDeep2 miRNA abundance quantification. Mean abundances between biological replicates (A,B) and fold change between conditions (C) were calculated with DEseq2 on the count tables output by each software package. Low abundance miRNAs with fewer than 2,000 counts across all samples are marked as transparent. Shown in pink are the miRNAs validated by qPCR in Figure 4. (A) Log scaled comparison of 451Lu-Par normalized miRNA counts of TEsmall versus miRDeep2, with a correlation coefficient of r = 0.882. (B) Log scaled comparison of 451Lu-BR miRNA counts of TEsmall versus miRDeep2, with a correlation coefficient of r = 0.910. (C) Comparison of log2 fold change as reported by TEsmall and miRDeep2, with a correlation coefficient of r = 0.867. [file Image_2.JPEG]
