# Supplementary material for: TEsmall Identifies Small RNAs Associated With Targeted Inhibitor Resistance in Melanoma
Source: Front Genet. 2018 Oct 5;9:461. doi: 10.3389/fgene.2018.00461 (PMC6186986; doi:10.3389/fgene.2018.00461)
Supplement: FILE S4 — Instructions for creating new annotation files for additional species. Instructions for customizing the path for stored annotation files. [file Table_4.DOCX]

**Supplemental File 4**

Currently only the annotated human, mouse and fly genomes are available for immediate use by TEsmall at the following location:

<http://labshare.cshl.edu/shares/mhammelllab/www-data/TEsmall/>

However, the user may provide their own genome and associated annotations by adding them to the hidden .tesmall directory in one’s home directory after installation (default location). The user must provide the genome of interest and rDNA in two fasta files, indices of these fasta files in the form of a fa.fai, and their associated bowtie indices. Annotations must be provided as .bed files with prefixes of exon, hairpin, intron, miRNA, piRNA_cluster, structural_RNA, and TE for each respective category. Examples can be found at the above link where genome info is provided for the currently supported genomes. We recommend the user take care to ensure there is no overlap of annotation species between categories as this will skew annotation by TEsmall. The directory of your genome should be structured as follows where “your_genome” represents how you would like to access your genome when calling TEsmall:

for all associated .bed files:

~/.tesmall/genomes/your_genome/annotation/*.bed

for all bowtie indices:

~/.tesmall/genomes/your_genome/sequence/bowtie_index/*.ebwt

for all genomic .fa and .fai files:

~/.tesmall/genomes/your_genome/sequence/

To change the default location of the tesmall annotation directories, one can simply set a “TESMALLROOT” environment variable that points to the new path when running TEsmall:

$ source activate tesmall

$ export TESMALLROOT=/your/desired/directory

$ tesmall -f Parental_1.fastq.gz DroKO_1.fastq.gz -l Parental DroKO

$ source deactivate
